# The influence of sea ice, wind speed and marine mammals on Southern Ocean ambient sound

**DOI:** 10.1098/rsos.160370

**Published:** 2017-01-11

**Authors:** Sebastian Menze, Daniel P. Zitterbart, Ilse van Opzeeland, Olaf Boebel

**Affiliations:** 1 Alfred Wegener Institute Helmholtz Centre for Polar and Marine Research, Bremerhaven, Germany; 2 Institute of Marine Research, Bergen, Norway; 3 Applied Ocean Physics and Engineering, Woods Hole Oceanographic Institution, Woods Hole, MA, USA; 4 Biophysics Group, Department of Physics, University of Erlangen-Nürnberg, Erlangen, Germany

**Keywords:** ocean ambient sound, ocean ambient noise, sea ice, Southern Ocean, Antarctic marine mammals, passive acoustic monitoring

## Abstract

This paper describes the natural variability of ambient sound in the Southern Ocean, an acoustically pristine marine mammal habitat. Over a 3-year period, two autonomous recorders were moored along the Greenwich meridian to collect underwater passive acoustic data. Ambient sound levels were strongly affected by the annual variation of the sea-ice cover, which decouples local wind speed and sound levels during austral winter. With increasing sea-ice concentration, area and thickness, sound levels decreased while the contribution of distant sources increased. Marine mammal sounds formed a substantial part of the overall acoustic environment, comprising calls produced by Antarctic blue whales (*Balaenoptera musculus intermedia*), fin whales (*Balaenoptera physalus*), Antarctic minke whales (*Balaenoptera bonaerensis*) and leopard seals (*Hydrurga leptonyx*). The combined sound energy of a group or population vocalizing during extended periods contributed species-specific peaks to the ambient sound spectra. The temporal and spatial variation in the contribution of marine mammals to ambient sound suggests annual patterns in migration and behaviour. The Antarctic blue and fin whale contributions were loudest in austral autumn, whereas the Antarctic minke whale contribution was loudest during austral winter and repeatedly showed a diel pattern that coincided with the diel vertical migration of zooplankton.

## 1. Introduction

Underwater ambient sound is created by the superposition of sounds from countless abiotic, biotic and anthropogenic acoustic sources; it is also termed the ‘acoustic environment’ [1] or ‘ambient noise’ [2]. This superposition renders it difficult to distinguish individual sound sources; however, ambient sound spectra can be used to study the different sound source types present within an environment and provide insights into the quality of an acoustic environment (e.g. potential masking effects due to anthropogenic noise).

In the ocean, sea surface processes, involving waves, wind stress, sea ice, precipitation and increasingly shipping form the chief sources contributing to ambient sound [2–4]. Wind stress is one of the major sources of ambient sound; the complex relation between wind speed and ambient sound varies with frequency and is strongest for frequencies above 500 Hz [2]. For the Pacific, Atlantic, Indian and Arctic Oceans, a growing body of literature reports increasing underwater sound levels caused by shipping and seismic exploration [5–9]. The Southern Ocean, on the other hand, is an acoustically pristine habitat due to its long distance from major shipping lanes and generally low levels of human activity. Anthropogenic sound sources rarely enter this region, mainly comprising sporadic research vessels and cruise ships that primarily target the Western Antarctic Peninsula area. The scarcity of such acoustically pristine habitats makes these invaluable in the context of passive acoustic monitoring (PAM) studies, as they can be used as a potential reference for trends in ocean ambient sound and to assess the natural variability of ambient sound. Nevertheless, only limited literature exists on the ambient sound conditions and trends in the Southern Ocean. Here, we analyse the temporal and spectral variation of Southern Ocean ambient sound over a 3-year period and discuss its relation to environmental factors such as wind speed and sea ice.

In regions closer to anthropogenic sound sources, evidence is accumulating that marine mammal communication, among other behaviours, is likely to be affected by increased ambient sound levels, particularly for baleen whale populations that are thought to rely on long-distance (low-frequency) communication [10–12]. Knowledge about the ambient sound marine mammals encounter in (and contribute to) the Southern Ocean's acoustic environment is limited and can provide valuable insights on how marine mammals interact with their acoustic environment in the relative absence of anthropogenic sound sources. Furthermore, multi-year passive acoustic records are important sources of year-round information on marine mammal distribution and behaviour. The Southern Ocean is thought to support more than 50% of the world's marine mammals in terms of biomass, many of which species have been subject to extensive exploitation [13]. Monitoring population recovery by means of visual surveys limits investigations to the austral summer months, when most regions in the Southern Ocean are accessible to vessels. PAM studies using autonomous recording units do not exhibit this seasonal bias [14]. For the Southern Ocean, various PAM applications have resulted in important findings, e.g. with respect to migration and distribution [15–17]. In addition to describing the ambient sound conditions in an important marine mammal habitat, we discuss how the vocal presence of the various marine mammal species relates to their spatio-temporal distribution and behaviour.

## 2. Material and methods

### 2.1. Moored recorders

**Figure 1.**
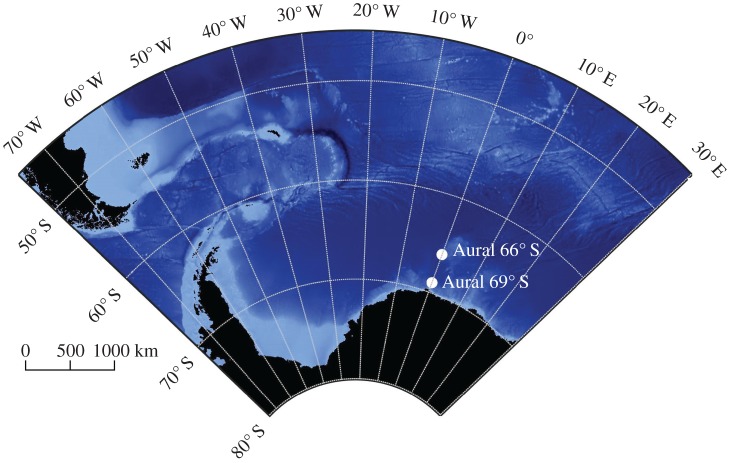
Map of mooring locations and 1 arc-min global relief model (ETOPO1) bathymetry [19].

Two autonomous underwater acoustic recorders (AURAL-M2, Autonomous Underwater Recorder for Acoustic Listening-Model 2, Multi-Électronique Inc.) were moored in the Atlantic sector of the Southern Ocean from March 2008 to December 2010 at 66°01′ S and 00°05′ E (Mooring ID: AWI-230-6) and at 69°00′ S and 00°00′ E (Mooring ID: AWI-232-9) [18]. Hereinafter, they will be identified as Aural 66° S and Aural 69° S. The mooring positions are shown in figure 1. Water depth at Aural 66° S was 3578 m, with the recorder moored at a depth of 260 m. Aural 69° S was moored 217 m deep in 3420 m deep waters. Permission to conduct fieldwork and deploy moorings in the Southern Ocean was granted by the German federal environmental agency (UBA permit number I 2.4-94003-3/207). The moorings consisted of Dyneema rope and carried multiple oceanographic devices. Details about the mooring set-up can be found in the electronic supplementary material, figures S1 and S2. Using a train wheel bottom weight and glass floats attached in regular intervals, an upright position of the moorings was achieved. Recorder depths varied within 2 m for Aural 66° S and 5 m for Aural 69° S due to currents shearing the moorings from their upright position.

Shear currents can induce strumming or flow noise into underwater acoustic recordings. In our recordings flow and strumming noises could not be discerned from manual screening of the 5-min spectrograms and power spectra, and were consequently assumed to have negligible impact on the results of this study. However, the shear current occasionally induced impulsive shackle noise at 69° S that contaminated some recordings between May and August in 2008 and June and October in 2009. The shackle noise could easily be identified by characteristic peaks between 40 and 60 Hz and occurred in so few recordings that we choose to not remove them from the dataset. Other than being occasionally visible as peaks in the long-term spectrogram and 50th and 95th percentile spectra of Aural 69° S, the shackle noise did not impact the results of this study.

The recorders were equipped with HTI-68-MIN (High Tech Inc.) hydrophones with a factory calibrated sensitivity of −164.6 dB re 1 V µPa^−1^. The self-noise of the hydrophones was reported by the manufacturer as 54 dB re 1 µPa^2^ Hz^−1^ at 10 Hz and 42 dB re 1 µPa^2^ Hz^−1^ at 100 and 1000 Hz. According to the Aural's manufacturer, the recorder's electronic self-noise is flat within ±1 dB over the usable frequency range from 10 Hz to 15 kHz [20]. Assuming an electronic broadband self-noise of 10 bits (typical value according to the manufacturer) in addition to the hydrophones' self-noise, results in a recorder self-noise of 55.0 dB re 1 µPa^2^ Hz^−1^ at 10 Hz and 45.4 dB re 1 µPa^2^ Hz^−1^ at 100 and 1000 Hz. In addition, the spectra measured by both recorders contained narrow peaks (approx. 3 Hz wide) between 80 and 2000 Hz caused by additional electronic noise. They remained constant over time and can be easily identified in the spectra.

The Aural's system gain was set to 22 dB, resulting in a saturation sound pressure level of 149 dB re 1 µPa. The recorded ambient sound spectra never reached saturation values. The sound level calibrations are solely based on factory calibration, no further pre- or post-calibration was performed, nor did we apply any frequency-specific correction of hydrophone sensitivity. According to the manufacturer the recorders' frequency response is flat within ±1 dB over the usable frequency range [20]. Adding an electronic error of 10 bits (typical value according to the manufacturer) to the 1 dB pressure error, the recorders' total error becomes ±61.6 µPa. On the decibel scale this will amount to a change of ±1.56 dB re 1 µPa^2^ Hz^−1^ for a 50 dB re 1 µPa^2^ Hz^−1^ signal and ±0.05 dB re 1 µPa^2^ Hz^−1^ for a 80 dB re 1 µPa^2^ Hz^−1^ signal. The recorders used UTC time and were set to record with a sample rate of 32 768 Hz for 5 min every 4 h starting 00.00 h daily, resulting in 486 h of acoustic recordings. As a gross of the recorded spectra hit the systems noise floor above 10 kHz, we limited our analysis to ambient sound between 10 Hz and 10 kHz. Data were stored losslessly in 16 bit wav files. Owing to internal data handling problems with the recorder, every 48th file was lost [21]. Additional parameters of the recorders are listed in table 1.

**Table 1.** Properties of the deployed AURAL-M2 recorders.

combined recording period	11 Mar 2008–16 Dec 2010
recording period or Aural 66° S	8 Mar 2008–16 Dec 2010
recording period or Aural 69° S	11 Mar 2008–21 Dec 2010
position	66°01.13′ S 000°04.77′ E and 68°59.74′ S 000°00.17′ E
sample rate	32 768 Hz
bit depth	16 bit
sampling scheme	5-min recordings every 4 h
frequency range	10–16 384 Hz
dynamic range	59–149 dB re 1 µPa

### 2.2. Ambient sound spectra and marine mammal contributions

**Figure 2.**
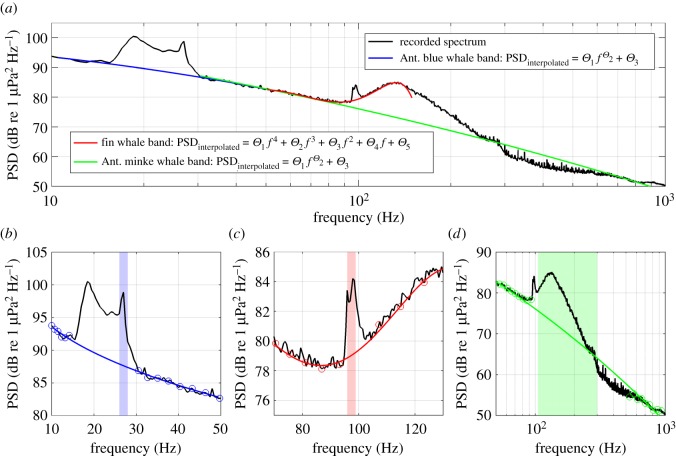
Example ambient sound spectrum with MMCs (visible as peaks) from Antarctic blue, Antarctic minke and fin whales and the respective interpolation functions used to calculate the non-MMC PSD. The black lines represent the measured PSD (at 66° S on 25 May 2008 12.00 h). (*a*) The three fitted interpolation functions in blue (Antarctic blue whale band), red (fin whale band) and green (Antarctic minke whale band). (*b*–*d*) Details of the measured spectra and interpolation function for each of the mentioned MMCs. The data points used to fit the interpolation functions to the measured spectra are displayed as circles and the frequency bands used to calculate the PSD_MMC_ are shaded in blue, red and green. In the interpolation functions, *θ_i_* represents the parameters that were fitted to each spectrum; PSD_dB_, the power spectral density in dB re 1 µPa^2^ Hz^−1^ and *f*, the frequency in Hertz.

All data processing and analysis was performed using Matlab 2015a. As typical for ambient sound analysis, the power spectral density (PSD) was calculated using a pre-set averaging length, which was set to the full 5 min of each recording. The PSD was calculated and averaged after Welch's method [22] using a window length of 2 s, 50% overlap and a fast Fourier transform (FFT) size of 2 s. Using this approach to calculate the spectrum has the advantage of biasing away from transient sounds (such as nearby marine mammal call trains or sea-ice cracks) and towards the quasi-continuous ambient sound. The resulting spectra showed persistent peaks between 15 and 30 Hz as well as 90 and 1000 Hz that occurred annually. These peaks represent the local ‘chorus-like’ cumulative sound energy produced by different marine mammal species. Marine mammal vocalizations are transient sounds, but the local combined sound energy of a group or population vocalizing during extended periods adds up to a quasi-continuous sound signal that can dominate the underwater ambient sound over certain frequency bands. These parts of the acoustic environment are further referred to as *marine mammal contributions* (MMCs). Peaks in the spectra (MMCs) could be assigned to different species by manual perusal of the 5-min recordings and comparison with published records of marine mammal vocal repertoires. The following species' contribution could be detected in the PSD dataset (table 2): Antarctic blue whales (*Balaenoptera musculus intermedia*) [23,24], fin whales (*Balaenoptera physalus*) [23,24], Antarctic minke whales (*Balaenoptera bonaerensis*) [25] and leopard seals (*Hydrurga leptonyx*) [26]. The numbers after each species indicate the key references used to identify the vocalizations in our recordings and MMC peaks in the ambient sound spectra. Figure 2 shows an example spectrum (black line) with peaks that represent the contribution of Antarctic blue, Antarctic minke and fin whale vocalizations to the ambient sound. The frequency ranges each MMC covered were determined by measuring the width of the respective peaks, and the core frequency bands (*f*_min_ and *f*_max_) that best characterized each MMC were chosen manually to avoid interference between the different MMCs (table 2). Both Antarctic blue and fin whales vocalize between 15 and 25 Hz, thus the Antarctic blue whale contribution was best classified using the narrow peak between 26 and 28 Hz and fin whale contribution with the peak between 96 and 99 Hz. Owing to the overlapping frequency ranges of the MMCs, the PSD that each species contributed to ambient sound (PSD_MMC_) was only calculated over each MMC's core frequency band (*f*_min_ to *f*_max_, column 3 in table 2) and not each MMC's entire frequency range (column 2 in table 2). Thus, all PSD_MMC_ values presented in this study represent the band-limited and not the total broadband sound energy each species contributes to ambient sound.

**Table 2.** Spectral range and interpolation functions used to calculate PSD_MMC_. In the interpolation functions *θ_i_* represents the parameters that were fitted to each spectrum PSD_dB_, the power spectral density in dB re 1 µPa^2^ Hz^−1^ and *f*, the frequency in Hertz.

species	frequency range of MMC spectral peak	core frequency range used to calculate PSD_MMC_ (*f*_min_ − *f*_max_)	interpolation function	frequency bands used to fit the interpolation function	correlation coefficient *r*^2^ between interpolated and measured PSD_dB_ during MMC absence	average correlation coefficient *r*^2^ between interpolated and measured PSD_dB_ in the frequency bands used to fit the interpolation function
Antarctic blue whales (*Balaenoptera musculus intermedia*)	15–28 Hz	26–28 Hz	power function: $\text{PS}{\text{D}}_{\mathrm{dB}}=\theta_{1}\;f^{\theta_{2}}+\theta_{3}$	10–15 and 30–50 Hz	—	0.97
fin whales (*Balaenoptera physalus*)	15–30 Hz and 97.5–98.5 Hz	96–99 Hz	4th degree polynomial: $\text{PS}{\text{D}}_{\mathrm{dB}}=\theta_{1}f^{4}+\theta_{2}f^{3}+\theta_{3}f^{2}+\theta_{4}f^{1}+\theta_{5}$	50–95 and 101–150 Hz	0.99	0.92
Antarctic minke whales (*Balaenoptera bonaerensis*)	100–1000 Hz	105–300 Hz	power function: $\text{PS}{\text{D}}_{\mathrm{dB}}=\theta_{1}\;f^{\theta_{2}}+\theta_{3}$	30–97 and 500–1000 Hz	0.72	0.98
leopard seals (*Hydrurga leptonyx*)	300–400 Hz	320–350 Hz	same as used for the Antarctic minke whale band	same as used for the Antarctic minke whale band	0.95	same as used for the Antarctic minke whale band

For each recording, the PSD_MMC_ in the four MMC core frequency bands was calculated by subtracting the estimated PSD without MMCs from the measured PSD. The hypothetical spectrum without the MMC (PSD_interpolated_) was calculated by fitting an interpolation function to the measured PSD around each MMC frequency band (coloured lines in figure 2). The frequency ranges and interpolation functions used for each MMC are specified in table 2. The interpolation functions with the best fit to the MMC's frequency band were chosen manually for each MMC, and differed between species due to the different shape of the spectrum at the different MMC bands. For the Antarctic blue whale, Antarctic minke whale and leopard seal bands, power functions best represented the non-MMC spectrum. A polynomial function provided the best fit for the fin whale band due to the nearby Antarctic minke whale peak. To characterize how well the interpolated PSD represented the non-MMC part of the ambient sound, the fit between PSD_interpolated_ and PSD_measured_ was calculated for periods of MMC absence (electronic supplementary material, figure S3). For the fin whale frequency band, the correlation coefficient *r*^2^ between PSD_interpolated_ and PSD_measured_ was 0.99, for the Antarctic minke whale band 0.72 and for the leopard seal band 0.95. As the Antarctic blue whale MMC was present year-round, we could not calculate the correlation coefficient between PSD_interpolated_ and PSD_measured_, but manual inspection of the fitted spectra confirmed that the interpolation function provided robust estimates of the non-MMC spectrum. The average correlation coefficient *r*^2^ between PSD_interpolated_ and PSD_measured_ in the frequency bands used to fit the interpolation function (coloured circles in figure 2) was higher than 0.9 for all interpolation functions (table 2). Using interpolation functions to determine the non-MMC part of the ambient sound for each recording avoids biases that could arise from the temporal variation in abiotic ambient sound. To quantify the contribution of the different species (PSD_MMC_), we averaged PSD_measured_ and PSD_interpolated_ over each MMC's respective core frequency band (table 2 and shaded areas in figure 2) and subtracted them from each other following

2.1 $$\begin{array}{rl} & {\text{PSD}}_{\mathrm{MMC}}=10\;{\text{log}}_{10}\left(\frac{\sum_{i=f_{\min}}^{f_{\max}}{\text{PSD}}_{\mathrm{measured}}}{n_{f_{\min}-f_{\max}}}-\frac{\sum_{i=f_{\min}}^{f_{\max}}{\text{PSD}}_{\mathrm{interpolated}}}{n_{f_{\min}-f_{\max}}}\right), \end{array}$$

where PSD_MMC_ is the PSD of each species contribution to ambient sound in the four MMC core frequency bands in dB re 1 µPa^2^ Hz^−1^, PSD_measured_ and PSD_interpolated_ are PSD values in μPa^2^ Hz^−1^, *f*_min_ and *f*_max_ are the respective boundaries of each frequency band (table 2) and $n_{f_{\min}-f_{\max}}$ the sample size between *f*_min_ and *f*_max_. The sample size was smallest for the Antarctic blue whale core frequency band ($n_{f_{\min}-f_{\max}}=5$) and largest for the Antarctic minke whale core frequency band ($n_{f_{\min}-f_{\max}}=382$). The PSD_MMC_ can only be measured when the MMC spectral peak is discernible in the power spectrum (i.e. when the MMC is louder than other sound sources in the respective frequency band). This can be quantified as signal-to-noise ratio (SNR) using

2.2 $$\begin{array}{rl} {\text{SNR}}_{\mathrm{MMC}} & =10\;{\text{log}}_{10}\left(\frac{\sum_{i=f_{\min}}^{f_{\max}}{\text{PSD}}_{\mathrm{measured}}}{n_{f_{\min}-f_{\max}}}\right)\\ & \;-10\;{\text{log}}_{10}\left(\frac{\sum_{i=f_{\min}}^{f_{\max}}{\text{PSD}}_{\mathrm{interpolated}}}{n_{f_{\min}-f_{\max}}}\right). \end{array}$$

To ensure a rigid analysis, an MMC was defined as present when its SNR_MMC_ was higher than the pre-defined threshold of 1 dB.

### 2.3. Wind speed and sea-ice data

The zonal and meridional wind speed fields were extracted from the European Centre for Medium-Range Weather Forecasts interim climate reanalysis dataset (ERA-interim) [27]. The temporal resolution of the fields was selected to 12 h (at 00.00 and 12.00 h) and the spatial resolution as 0.25°.

Gridded sea-ice concentration data were obtained from the University of Bremen, based on their analysis of data from the Advanced Microwave Scanning Radiometer Earth Observing System (AMSR-E) [28]. The data consist of daily average values on a polar stereographic grid with a spatial resolution of 6 × 4 km. To correlate the gridded datasets with the two ambient sound time series, we calculated the average values of concentric circles in 50 km radius steps for each mooring location.

The Antarctic sea-ice extent time series was obtained from the National Snow and Ice Data Centre [29]. Sea-ice draught was measured by an upward looking sonar installed in the same mooring as Aural 69° S and the error-corrected sea-ice draught data obtained from the PANGEA database [30]. All correlation coefficients *r* in this study were calculated using Pearson's method [31].

## 3. Results

### 3.1. Spectra and band levels

**Figure 3.**
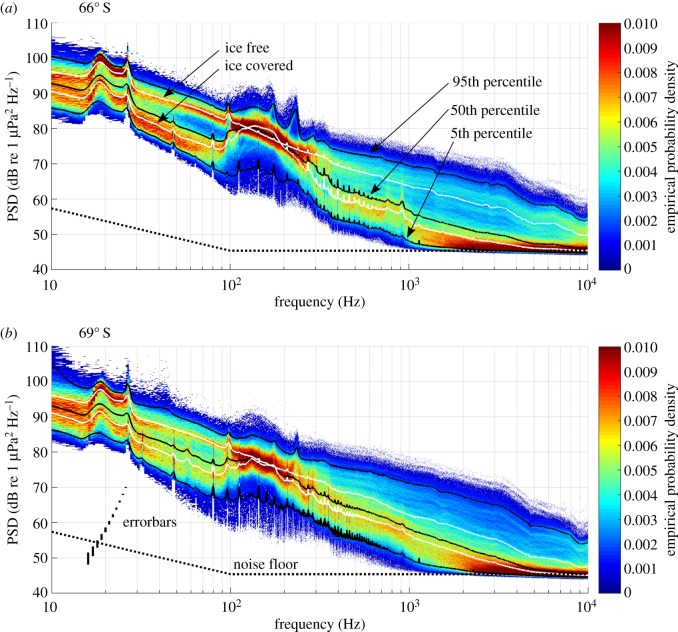
Spectral statistics at (*a*) 66° S and (b) 69° S (as based on factory calibration). Colour shows empirical spectral probability density as a function of frequency and spectral levels, the black solid lines the 5th, 50th and 95th percentile spectral levels and the white lines the average spectra during ice-covered (mean sea-ice concentration in 200 km radius > 50%) and ice-free conditions (mean sea-ice concentration in 200 km radius < 50%). The dashed lines show the system's noise floor. The vertical bars in (*b*) illustrate the error bars (±61.6 µPa) for signals from 50 to 70 dB re 1 µPa^2^ Hz^−1^.

The recorded spectral probability density over frequency is shown in figure 3. At frequencies above 1000 Hz, large parts of the spectra, visible as red clusters in the spectral probability density, met the recorders' noise floor. Between 15 and 100 Hz, the spectral probability density shows a bimodal distribution (two separated (red) areas of increased probability) that can be linked to the sea-ice conditions. The two white lines represent the average spectrum during ice-free and ice-covered periods and match the bimodal distribution. Neglecting the noise floor, the median, 5th and 95th percentile spectra are similar to a power law spectrum. However, peaks associated with marine mammal vocalizations (MMCs, table 2) can be found between 15 and 30 Hz as well as 90 and 1000 Hz. The spectra, furthermore, show narrow peaks (approx. 3 Hz wide) related to recorder internal electronic noise, which have a narrower bandwidth than the MMC peaks and stay constant over time.

**Figure 4.**
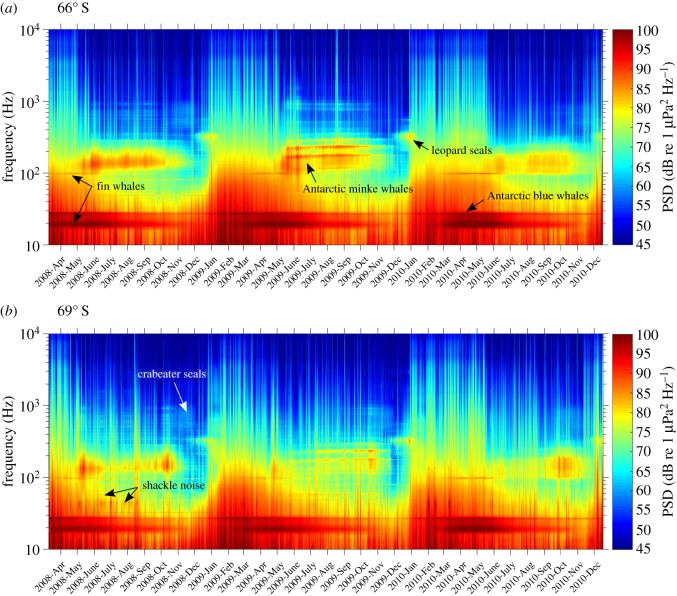
Long-term spectrograms of recordings from 66° S (*a*) and 69° S (*b*). PSD based on factory calibration.

The 3-year time series of PSDs, referred to as long-term spectrograms hereinafter, are displayed in figure 4. The temporal variation of the spectra follows a seasonal cycle, where the highest spectral levels occur in austral summer, between January and March, followed by a gradual decrease in spectral levels that can be associated with the growing sea-ice cover. In the long-term spectrogram from 69° S (figure 4*b*), the sound generated by moving shackles can be seen as faint dotted line at 40 and 57 Hz in 2008 and 2009.

### 3.2. Marine mammal contributions

**Figure 5.**
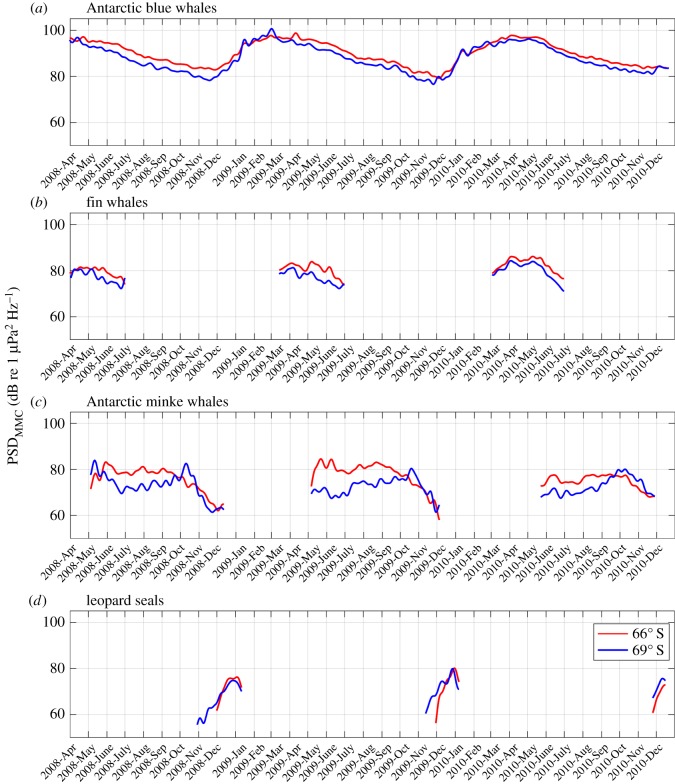
Comparison of marine mammal contribution PSD time series (low-pass filtered with a 7-day window Butterworth filter) between 66° S (red) and 69° S (blue) (as based on factory calibration): (*a*) Antarctic blue whale contribution (*Balaenoptera musculus intermedia*), (*b*) fin whale contribution (*Balaenoptera physalus*), (*c*) Antarctic minke whale contribution (*Balaenoptera bonaerensis*) and (*d*) leopard seal contribution (*Hydrurga leptonyx*). The PSD_MMC_ is only plotted where the SNR_MMC_ is above 1 dB. Note that PSD_MMC_ was averaged over different frequency bands: 26–28 Hz for Antarctic blue whales, 96–99 Hz for fin whales, 105–300 Hz for Antarctic minke whales and 320–350 Hz for leopard seals (table 2).

The long-term spectrograms exhibit annually reoccurring horizontal lines between 15 and 30 Hz as well as 90 and 1000 Hz (figure 4, indicated by arrows), which intensify in amplitude seasonally. They are associated with marine mammal vocalizations and represent the local cumulative sound energy of all individuals of a species producing a specific call type. We analysed the temporal and spatial variation of PSD_MMC_ contributed by Antarctic blue, fin and Antarctic minke whales and leopard seals in their respective frequency bands (figure 5). The sound energy emitted by the characteristic broadband vocalizations of crabeater seals (*Lobodon carcinophaga*) was occasionally present in the long-term spectrograms between 500 and 1000 Hz (figure 4) [32], but was too faint for a robust analysis.

#### 3.2.1. Antarctic blue whales

Between 27 and 28 Hz, the most persistent peak in the long-term spectrograms (figure 4) is associated with Antarctic blue whale vocalizations. The recorded Antarctic blue whale contribution consists of so-called Z-calls, comprising three components [33], which are emitted between 18 and 27 Hz [24]. Owing to interference with fin whale vocalizations around 20 Hz and the remarkable stereotypy of the upper call component (Z-call), the Antarctic blue whale contribution is best represented by band levels between 26 and 28 Hz [34]. The blue whale contribution was recorded continuously, reaching highest PSD_MMC_ values in between February and June and lowest between September and December (figure 5*a*). Antarctic blue whale PSDs were similar at 66° S and 69° S from January to March and louder at 66° S compared with 69° S for the rest of the year.

#### 3.2.2. Fin whales

Fin whales in the Southern Ocean emit pulsed calls with main energy around 20 and 89 or 99 Hz, depending on region [17,23]. In the long-term spectrograms (figure 4), the upper fin whale call component forms a narrow peak at 98 Hz, which is 9 Hz higher than measurements from fin whales off the West Antarctic Peninsula [23]. To exclude interference with Antarctic blue whale vocalizations, the PSD of the Antarctic fin whale contribution is best represented by the upper call component at 98 Hz. The Antarctic fin whale contribution was present (SNR > 1 dB in the 96–99 Hz band) each year between March and July. Fin whale PSDs were generally louder at 66° S than 69° S, especially from April to July (figure 5*b*).

#### 3.2.3. Antarctic minke whales

One of the most distinct patterns in the long-term spectrograms is observed between 100 and 300 Hz, reoccurring between May and November each austral winter (figure 4). It is associated with Antarctic minke whale vocalizations [25]. The PSDs of the Antarctic minke whale contribution differed by up to 10 dB between the two recording locations, with the contribution being louder at 66° S most of the year (figure 5*c*). At 66° S, Antarctic minke whale PSDs were highest between May and September, followed by a decrease in October and November. At 69° S, Antarctic minke whale PSD increased until October, followed by a sharp decrease in November. An exception to this pattern occurred in 2008, where Antarctic minke whale PSDs were stronger at 69° S compared with 66° S in the beginning of May, and increased at 66° S and decreased at 69° S until June. In the long-term spectrograms, the frequency characteristics of the Antarctic minke whale contribution varied from year to year (figure 4). However, throughout the recording period, the major part of the received Antarctic minke whale sound energy remained between 100 and 300 Hz.

**Figure 6.**
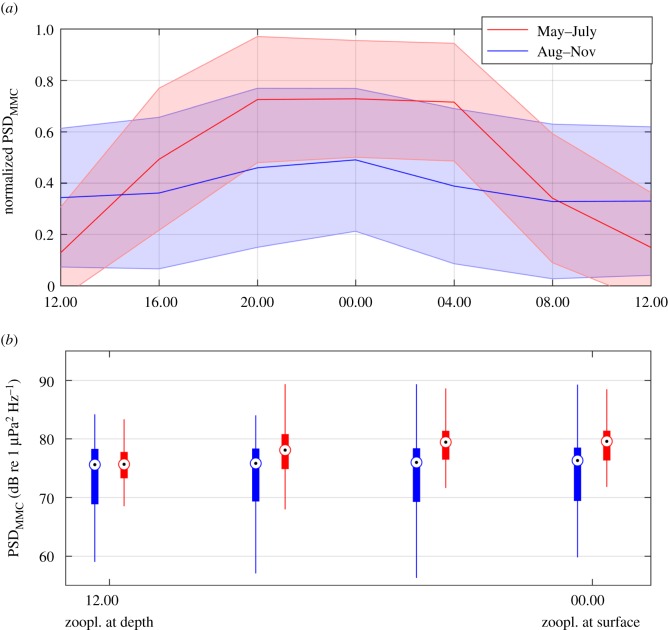
Seasonal presence of diel pattern in the Antarctic minke whale contribution in relation to the diel vertical migration (DVM) of zooplankton. (*a*) The averaged Antarctic minke whale PSD, normalized between 0 and 1 for each day, over the hour of day. Red represents the period between 1 May and 31 July, blue the period between 1 August and 30 November. Bold lines represent the average normalized PSD and shaded areas the standard deviations. (*b*) The relationship between Antarctic minke whale PSD (as based on factory calibration) and an idealized DVM pattern (zooplankton at depth during midday and at surface during midnight). The horizontal axis represents the idealized vertical position of zooplankton and the boxes Antarctic minke whale PSD averages for four vertical zooplankton positions (related to time of day). Points represent median values, thick lines 25th and 75th percentiles and thin lines the minimum and maximum values. Colour indicates the time periods corresponding to (*a*). The figure indicates that the timing of Antarctic minke whale vocal activity and DVM of zooplankton are connected during winter (polar night).

On a much shorter time scale, the Antarctic minke whale acoustic contribution followed a diel cycle from the end of April to the beginning of August each year. Figure 6*a* displays the distribution of Antarctic minke whale PSD over a 24 h cycle (starting and ending at 12.00 UTC) at 66° S, normalized for each day between 0 and 1. The diel cycle's phase remained stable throughout the recording period, with Antarctic minke whale PSDs being louder at midnight than midday. The diel cycle was strongest in austral winter 2009 (electronic supplementary material, figure S4).

#### 3.2.4. Leopard seals

Antarctic seals contribute distinctly to the underwater acoustic environment of the Southern Ocean [26]. Leopard seals are particularly vocally active (high density of calls) and their calls contain energy at frequencies low enough to contribute peaks to the long-term spectrograms. Peak energy of the contribution is found between 320 and 350 Hz, partly overlapping in frequency (but not the core frequency band *f*_min_ to *f*_max_) with the Antarctic minke whale contribution (figure 4). The leopard seal contribution was loudest between December and January annually (figure 5*d*).

### 3.3. Relation to sea ice and wind speed

The impact of the physical environment on underwater sound levels was explored by comparing the average PSD in the frequency bands 30–80 and 500–1000 Hz with spatially averaged wind speed and sea-ice concentration as well as sea-ice draught and extent. These frequency bands were chosen to exclude interferences with the MMCs and to avoid the recorder noise floor. Both frequency bands show similar patterns over their respective bandwidth in the spectral probability density spectra (figure 3): a bimodal pattern related to sea ice for the 30–80 Hz band and a broad distribution for the 500–1000 Hz band, which indicates a differing response to the physical environment between the two bands.

**Figure 7.**
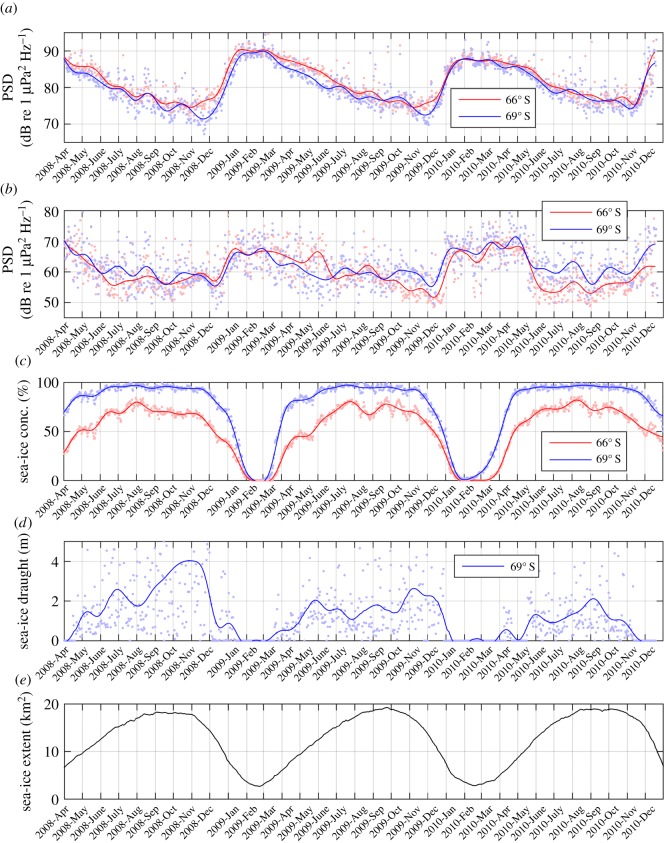
Comparison of ambient sound (as based on factory calibration) and sea-ice time series. (*a*,*b*) The average PSD of two frequency bands: 30–80 (*a*) and 500–1000 (*b*) Hz at both 66° S (red) and 69° S (blue). Each dot represents a 5-min recording, and the solid lines a 20-day running mean. (c) Spatially averaged sea-ice concentration (within 200 km radius) with the solid line representing a 20-day running mean. (*d*) The sea-ice draught at 69° S with the solid line representing a 20-day running mean. (*e*) The Antarctic sea-ice extent in square kilometres.

**Figure 8.**
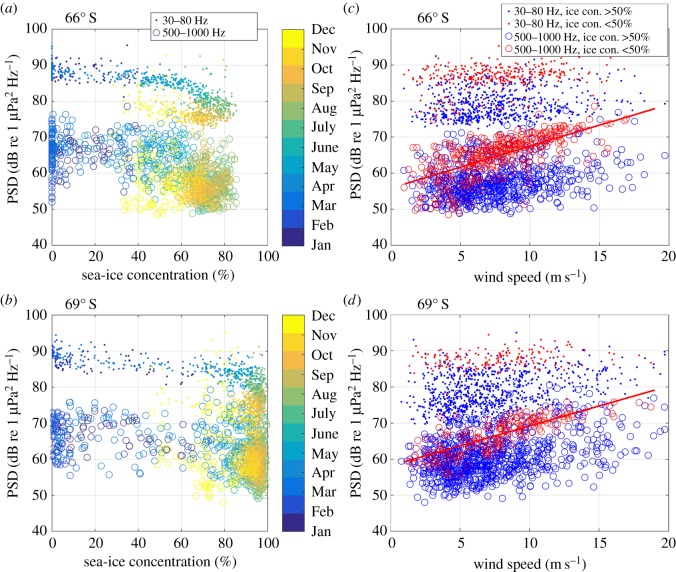
Relation between ambient sound levels (as based on factory calibration), wind speed and sea-ice concentration, each dot represents a 5-min recording. (*a*,*b*) The average PSD of two frequency bands (30–80 and 500–1000 Hz) over spatially averaged sea-ice concentration (within 500 km radius), with month represented by colour. (*c*,*d*) The average PSD of the two bands over spatially averaged wind speed (within 200 km radius), with ice-free conditions (sea-ice concentration smaller than 50%) represented by red markers and ice-covered conditions (sea-ice concentration larger than 50%) by blue markers. The red lines show a linear fit to the data points representing 500–1000 Hz PSD and wind speed under ice-free conditions. Average slope of linear fit was 1.13 and average correlation coefficient *r* was 0.7.

Figure 7 compares the time series of PSD in the two bands and spatially averaged sea-ice concentration (percentage of area covered by sea ice), sea-ice draught (thickness of the submerged sea-ice layer) and extent (total area covered by sea ice). A scatterplot of the relationship between spatially averaged sea-ice concentration (within 500 km radius) and PSD is shown in figure 8*a*,*b*, where each marker represents a 5-min recording and marker colour encodes the recording month. The time series and scatter plots indicate an inverse relationship between sea-ice concentration and PSD, which is clearest for the 30–80 Hz frequency band (dots in figure 8) and between February and July (blue hues in figure 8). Between August and November (yellow hues in figure 8), the PSD decreased even though the sea-ice concentration remained approximately constant, whereas between December and January, PSD and sea-ice concentration again showed an inverse relationship. For both frequency bands, the PSD continuously decreased throughout austral winter and reached lowest values in October and November, whereas the sea-ice concentration saturated already between June and August and the sea-ice extent reached its maximum in September and October (figure 7). Sea-ice draught (only measured at 69° S) increased continuously throughout austral winter and reached highest values around November. Throughout the observed period, the annual maximum sea-ice draught decreased, whereas the sea-ice concentration showed no such trend. The sea-ice extent increased, especially in the Weddell Sea between January and May [35]. In the 30–80 Hz band, the annual minimum PSD (in November and December) increases, whereas the annual maximum PSD (January to March) decreases from 2009 to 2010. At both locations, the correlation between sea-ice concentration and PSD was strongest in the 30–80 Hz band (electronic supplementary material, figure S5); however, the spatial averaging radius corresponding to the strongest correlation was 200 km at 66° S (*r* ≈ −0.8) compared with 2000 km at 69° S (*r* ≈ −0.9).

**Figure 9.**
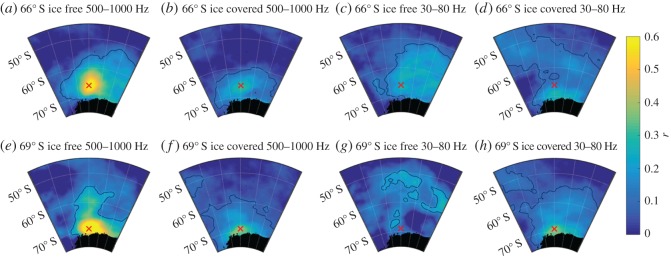
Maps showing the correlation between wind speed and average PSD in two frequency bands under ice-free (average sea-ice concentration in 200 km radius < 50%) and ice-covered (average sea-ice concentration in 200 km radius > 50%) conditions. The colour scale indicates the correlation coefficient *r* for each cell of the ERA-interim grid, the red cross marks each recorders location and the black contour lines encircles areas with *p* > 0.001, indicating a significant relation between PSD and wind speed. (*a*–*d*) Recordings from 66° S and (*e*–*h*) from 69° S. The correlation between average 500–1000 Hz PSD and wind speed (*a*,*e*) during ice-free conditions and (*b,f*) during ice-covered conditions. The correlation between average 30–80 Hz PSD and wind speed (*c*,*g*) during ice-free conditions and (*d*,*h*) during ice-covered conditions.

Figure 9 shows maps of the correlation between wind speed in each ERA-interim cell and PSD under different sea-ice conditions. During ice-free conditions (average ice concentration in 200 km radius < 50%), wind speed strongly correlates (*r* > 0.5) with 500–1000 Hz PSD within a 200 km radius around both recorders' locations (figure 9*a*,*e*), whereas average PSD between 30 and 80 Hz correlates poorly with local wind speed (figure 9*c*,*d*). However, at 66° S wind speed and 30–80 Hz PSD correlate weakly (*r* ≈ 0.3) over an area between 50° S and 70° S (figure 9*c*) during ice-free conditions. During ice-covered conditions (average ice concentration in 200 km radius > 50%), wind speed correlates weakly (*r* ≈ 0.3) with 500–1000 Hz PSD within a 600 km radius for Aural 66° S (figure 9*b*) and within an area roughly indicating the coastal polynya for Aural 69° S. For both recorders, the 30–80 Hz PSD and wind speed correlate weakly in an area roughly indicating the coastal polynya, during ice-covered conditions. The relationship between local wind speed (averaged within 200 km radius) was also analysed as scatterplot (figure 8*c*,*d*). The response to increasing wind speed is similar at both locations: in the 30–80 Hz band, the PSD shows no substantial trend with increasing wind speed, both during ice-free and ice-covered conditions. In the 500–1000 Hz band, on the other hand, the PSD increases with increasing wind speed during ice-free conditions (slope of linear fit ≈1.13, *r* = 0.7), and shows no substantial trend during ice-covered conditions.

## 4. Discussion

The recorded long-term spectrograms represent the prevailing natural sound conditions in the Atlantic sector of the Southern Ocean, consisting of the cumulative emissions from air–sea-ice interaction, marine mammals and icebergs or shelf ice (approx. 200 km to the south of Aural 69° S). Ambient sound generated by shipping typically covers a frequency range from 10 to 1000 Hz and would be difficult to distinguish from sea-surface-generated sound in the ambient sound spectra [2]. However, due to the recorders' large distance to major shipping lanes (over 4000 km), ambient sound generated by shipping is likely to be of minor importance for our observations [36]. Ship-generated sound only dominated the spectrum when RV *Polarstern* approached the moorings containing the recorders (i.e. for deployment and recovery of the moorings). We thus assume that local and distant sea surface processes are the major abiotic sources of ambient sound in the Southern Ocean.

In the PSD dataset, the recorders' self-noise occurred as constant narrow peaks between 80 and 2000 Hz and broadband noise at frequencies above 1000 Hz. As the self-noise remained constant over time and the shackle noise occurred at frequencies away from the MMC bands, the PSD_MMC_ measurements were not affected. Owing to the self-noise's low amplitude and scarcity of the shackle noise, we assume that both had negligible impact on our analysis of the relation between environmental parameters and PSD. It did, however, limit our analysis to frequencies below 1000 Hz, as most spectra above that hit the systems noise floor.

### 4.1. Sea-surface-generated ambient sound

#### 4.1.1. Wind stress

Wind stress at the sea surface generates sound between approximately 0.1 and 20 kHz [2,3]. The increase in sound levels with increasing wind speeds depends on wind speed and frequency and is largest for frequencies above 500 Hz [2]. Our observations show an approximately linear relationship (slope of linear fit ≈1.13, figure 8) between local wind speed (averaged within 200 km radius) and PSD under ice-free conditions in the 500–1000 Hz band, which is in accordance with other studies [2,4,37]. The large scatter in the relationship between local wind speed, sea ice and PSD can be attributed to the coarse sampling of the climate data (figure 8). The correlation between local wind speed and ambient sound levels (figures 8 and 9) is comparable with previous studies [38,39]. The low-frequency (10–500 Hz) spectrum is dominated by the cumulative sound emitted from distant sources and surface waves [2], which explains the poor correlation between local wind speed and ambient sound levels below 500 Hz (figures 8 and 9). The lack of correlation between local wind speed and ambient sound during the presence of sea ice confirms the expectation that the sea-ice cover effectively shields the sea surface from direct wind stress and prevents local wind-generated sound. Figure S6 in the electronic supplementary material illustrates the changing relation between PSD and local wind speed for the summer and autumn of 2009 by comparing the long-term spectrogram with the local wind speed.

#### 4.1.2. Local versus distant sound sources

South of the Antarctic Convergence Zone (i.e. south of 60° S), the sound speed minimum is located close to the sea surface, thus creating a surface sound duct [40]. Both recorders had been moored at the deeper end of this duct (electronic supplementary material, figure S7). According to Buckingham [41], under upward refracting conditions, ambient sound consists of a direct path (from local sources above the receiver) and a modal (mainly from distant sources) component. He, furthermore, inferred that sound levels decrease with depth, with the strongest gradients occurring in the modal component, in the upper 500 m and frequencies above 100 Hz [41]. However, as the depth difference between our recorders was only 43 m, we assume that the depth dependence of sound levels only had a minor impact on the observed PSD. In the 30–80 Hz band, Buckingham's theory predicts the dominance of the modal component, whereas in the 500–1000 Hz band, the theory predicts an overlap of the direct path and modal components [41].

Given that wind-induced surface motion is one of the major sound sources over the observed spectrum [2], the correlation maps between wind speed and PSD in figure 9 roughly indicate regions of significant noise contribution. The correlation maps indicate that during ice-free conditions, 500–1000 Hz PSD correlates strongly with local wind speed (approx. 200 km radius) and weakly with wind speed in a broad region around the recorder (approx. 600 km radius), whereas during ice-covered conditions 500–1000 Hz PSD only correlates weakly with wind speed in a broad region around the recorder (approx. 600 km radius). These results suggest that, in the 500–1000 Hz band, distant sources (modal component) dominate under ice-covered conditions, whereas local sources (direct path component) dominate under ice-free conditions. This is supported by the observation that the spatial averaging radius corresponding to the strongest correlation between sea-ice concentration and PSD was a magnitude larger at 69° S (surrounded by more sea ice) compared with 66° S (electronic supplementary material, figure S5). In the 30–80 Hz band, the correlation maps (figure 9) indicate the dominance of distance sources during both ice-free and ice-covered conditions. Under ice-covered conditions and for both frequency bands at 69° S, and for the 30–80 Hz band at 66° S, the correlation maps show highest values for a region indicative of the coastal polynya. This suggests that the polynyas around the Antarctic continent are important contributors of ambient sound during austral winter.

#### 4.1.3. Sea ice as sink and source of underwater sound

The comparison between PSD and sea-ice concentration, draught and extent showed that a growing ice cover decreases ambient sound levels across the observed spectrum, with the strongest correlation in the 30–80 Hz band (figures 7 and 8). Low-frequency ambient is generated by sea surface motion, through a combination of several mechanisms [4,42]. The sea ice effectively attenuates surface motion, and thus reduces low-frequency (10–500 Hz) sound generated by surface waves. In addition to attenuating the local sound source mechanism, an increase in sea-ice extent, concentration, roughness and thickness will increase the attenuation of sound from distant sources [43]. The hypothesis that sea-ice thickness and roughness are important variables determining under-ice sound levels is supported by the fact that minimum PSD values in the 30–80 Hz band are reached while sea-ice draught measurements reach maximum values (approx. November, figure 7), which is after the sea-ice concentration and sea-ice extent reach maximum values (approx. July and September, figure 7). The increase of the annual minimum PSD in the 30–80 Hz band corresponds to the decrease of the annual maximum sea-ice draught (figure 7), whereas the decrease of the annual maximum PSD in the 30–80 Hz band probably corresponds to an increase of the annual minimum sea-ice extent (January to March) in the Weddell Sea [35]. The observation that, during winter, PSD values in the 30–80 Hz band were slightly lower at 69° S than 66° S, can be attributed to higher concentrations of sea ice (and a larger surrounding sea-ice area) at 69° S compared with 66° S. During winter, the 500–1000 Hz band PSD is slightly higher at 69° S than at 66° S (figure 7). The correlation maps (figure 9) suggest that the extra sound energy originates from the marginal sea-ice zone and ice-free areas of the coastal polynya. Overall, the observed relation between ambient sound levels, sea-ice concentration and wind speed is comparable with the Arctic Ocean, where a 5–20 dB reduction of ambient sound levels was observed under sea ice [37].

In our observations, the net effect of the sea-ice cover is a reduction of ambient sound levels. However, sea ice is also a source of underwater sound, especially in the marginal sea-ice zone, where surface waves penetrate the ice floes [36,44]. Sea-ice-generated sound (icequakes) can contribute to ambient sound over the observed spectrum [2,36,44–46]. Icebergs and shelf ice can be intense sound sources (especially during calving events) and can contribute to ambient sound below 100 Hz [36,47]. Sound from the shelf ice edge (approx. 200 km from Aural 69° S and 500 km from Aural 66° S) could explain the increased PSD below 50 Hz at 69° S (figure 3). The effect of precipitation on ambient sound is not considered here, but has generally shorter and more localized effects on ambient sound than wind stress, and is of lesser importance than wind stress for the frequency bands analysed here (30–80 and 500–1000 Hz) [2].

### 4.2. Marine mammal contributions

**Figure 10.**
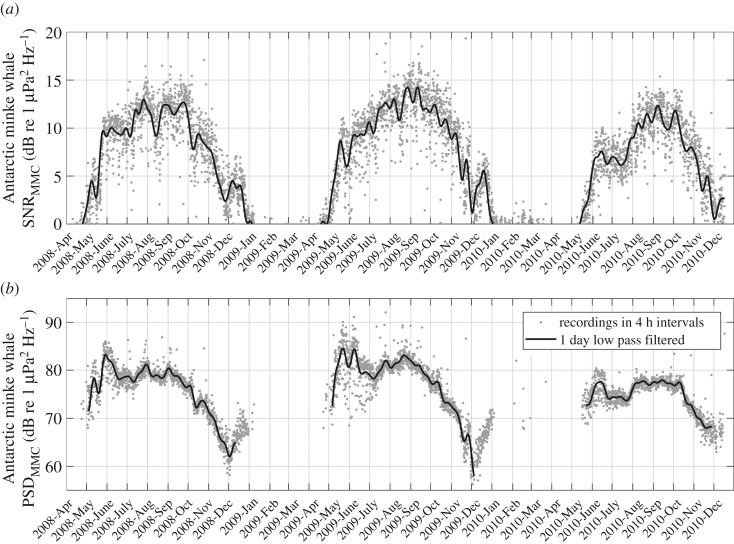
Comparison of the Antarctic minke whale contributions signal-to-noise ratio (SNR) and power spectral density (PSD, as based on factory calibration) recorded at 66° S. Grey dots represent values for each recording, black line the 1-day running mean. The PSD and SNR time series peak at different times and the May–June peak in PSD is absent from the SNR time series.

Previous studies on the contribution of marine mammals to underwater ambient sound used relative metrics to describe the contribution (acoustic power method in [23], fin whale index in [48] and blue whale index in [49]), effectively describing the SNR of MMCs (termed signal in this context) to the abiotic ambient sound (termed noise in this context). This approach carries the risk of adding interference from abiotic sound fluctuations into the MMC measure. To avoid this, we calculated the strength of the MMC using absolute PSDs by subtracting interpolated abiotic spectra from the measured spectra (see §2.2). To illustrate the difference between the two metrics, figure 10 displays the Antarctic minke whale contributions SNR_MMC_ and PSD_MMC_. The two time series exhibit different temporal patterns, peaking at different times. If the abiotic sound were roughly constant over time, the two time series would show similar patterns. But as the abiotic ambient sound shows substantial temporal variation, the SNR_MMC_ reflects both variation in the MMC (signal) and abiotic ambient sound (noise). To avoid this interference with abiotic sound fluctuation, we choose to analyse the MMCs using PSD_MMC_ as metric. Avoiding this interference is particularly relevant when sound energy contribution metrics are used to infer information on the animal's distribution and behaviour.

There are several other aspects that have to be borne in mind when deriving information on marine mammal occurrence and distribution from ambient sound spectra. The observed marine mammal PSDs only indicate periods of intense vocal activity, as sporadic calls will not be visible as peaks in the acoustic power spectra (too low SNR_MMC_). Furthermore, marine mammal PSDs only contain information about the marine mammal presence, not absence (animals can be present in the area but not vocalize) and the vocalizing population could be sex or age segregated. For a given location, increased PSDs_MMC_ could be caused by a combination of processes: an increase in number of vocalizing animals, increase in source level, increase in call rate, decreasing distance to the vocalizing animals or decreasing transmission loss between the vocalizing animals and the recorder.

The recorded PSDs_MMC_ show annually reoccurring patterns that vary between species and recorder location (figure 5). It is important to note here that the PSDs of each species' contribution were only calculated over each MMC's core frequency band (*f*_min_ to *f*_max_) and not each MMC's entire frequency range (table 2). Thus, a comparison of PSDs between different species or with abiotic sound sources is most informative when comparing relative patterns. The following sections discuss the species-specific observations.

#### 4.2.1. Antarctic blue whales

Blue whale calls are recorded in all the world's oceans and distinct call types have been associated with specific subpopulations [50]. A persistent decrease in blue whale vocalization frequency has been observed globally and was also found in our recordings [34,51]. In the North Pacific and Indian Ocean, the spatial and temporal variation in blue whale vocal activity has been associated with annual migration patterns [34,38,52]. The observed inverse relation between sea-ice extent and Antarctic blue whale PSD indicates either latitudinal migration of vocalizing Antarctic blue whales or changes in vocal activity related to the seasonal cycle or sea ice. During austral summer and its limited sea-ice cover (January–March), Antarctic blue whale PSD is similar at 66° S and 69° S, while during austral winter (with high concentrations of sea ice), Antarctic blue whale PSD is stronger at 66° S than 69° S. Along the western shelf of the Antarctic Peninsula, Širović *et al*. [23] observed two annual peaks (March–May and October–November) in Antarctic blue whale vocal activity. The first of these peaks coincides with the Antarctic blue whale PSD peak and the minimum in sea-ice extent in this study, whereas an October–November peak in vocal activity is absent in our data (figure 5*a*). Our observations match the Antarctic blue whale vocal activity pattern in the Drake Passage [36] and the Weddell Sea [49], where highest vocal activity was recorded in the end of summer and beginning of autumn (January–May). The 1996 stock of approximately 1700 Antarctic blue whales was estimated to be 0.7% the size of the pre-whaling stock (approx. 239 000 animals) [53]. Considering the sound produced by the contemporary population, the sound energy Antarctic blue whales contributed to the acoustic environment has probably been considerably higher before the depletion of stocks.

#### 4.2.2. Fin whales

Owing to their low frequency (approx. 20 Hz) and high call rate, the pulsed vocalizations of fin whales are detectable as a peak in many recorded ambient sound spectra [54–56]. West of the Antarctic peninsula, fin whale vocal activity peaks annually between March and May [17,23], whereas fin whale PSD in our observations peaks between March and June, similar to vocal activity observations from Eastern Antarctica (approx. 67° S, approx. 70° E) [17]. The upper frequency component of the fin whale contribution west of the Antarctic Peninsula and in the Scotia Sea [17,23] is 9 Hz below the one we observed (at 98 Hz), whereas the fin whale contributions measured near Eastern Antarctica match our recordings [17]. The spectral offsets between the different recording locations suggest separate populations, which confirms Širović's findings [17], and indicates a connection between fin whales recorded in the Eastern Antarctic and the Atlantic sector of the Southern Ocean. The varying difference in fin whale PSD between 66° S and 69° S could be an indicator of latitudinal migration and the stronger fin whale PSD at 66° S compared with 69° S indicates that vocal activity or migration are influenced by the seasonal cycle or sea ice. Although the major part of each fin whale vocalization's sound energy is contained in the lower call component (approx. 20 Hz) [17], we only analysed the variation of the upper call component between 96 and 99 Hz to avoid interference with the Antarctic blue whale contribution. Given that fin whales could vary their use of the two call components, the PSD corresponding to the lower call component might show a different pattern to the PSD we measured for the upper call component.

#### 4.2.3. Antarctic minke whales

Antarctic minke whale vocalizations have only recently been identified and were previously known as ‘Bioduck’ signals [25]. Studies along the Weddell Sea coast and near the Australian coast most frequently observed Antarctic minke whale vocal activity in austral winter, and no vocal activity in austral summer [57,58]. A similar pattern was found in our recordings (figure 5*c*). The difference in Antarctic minke whale PSD between 66° S and 69° S suggests latitudinal migration of vocalizing minke whales or local changes in vocal behaviour. A part of the annual and spatial variation of PSD could also be caused by changes in transmission loss due to sea-ice growth and melting. Annually, Antarctic minke whale PSD peaked during May and October at 66° S, and during October and December at 69° S (except a unique peak in PSD at 69° S in May and June 2008). This suggests a southward migration of vocalizing Antarctic minke whales, or southward shift in vocal behaviour in austral spring. Antarctic minke whales frequently feed on dense patches of krill under sea ice [59]; the variation in Antarctic minke whale PSD could thus be linked to favourable prey and sea-ice conditions [60].

A connection between feeding and vocal behaviour is supported by the finding that the Antarctic minke whale contribution followed a diel cycle from the end of April to August (figure 6; electronic supplementary material, figure S1), with high vocal activity at midnight and less at midday. For northern minke whales, a similar diel cycle in vocal activity was observed in Massachusetts Bay and associated with feeding and mating [61]. The period when the Antarctic minke whale population's vocal behaviour follows a diel pattern overlaps with the time of minimal irradiance and growing sea-ice extent in the Southern Ocean. The diel calling cycle is thus probably not linked to irradiance cues from the sun. However, the diel vertical migration of zooplankton (DVM), including Antarctic krill (*Euphausia superba*), has been shown to occur in the polar night in the Atlantic sector of the Southern Ocean [62]. The occurrence of DVM coincides with the diel pattern in Antarctic minke whale PSD in time and space. Acoustic Doppler current profilers moored along the Greenwich meridian observed distinct DVM patterns from February to October at 66° S and between February and June at 69° S [63,64]. This coincides with the timing of the observed Antarctic minke whale PSD diel pattern. As typical for most DVM patterns, high zooplankton concentrations are found at the surface at night (when the Antarctic minke whale PSD was loudest) [62,63], and low concentrations during the day (figure 6*b*). Antarctic minke whales have been observed feeding directly under the sea surface, skimming the underside of sea ice for krill [59]. Given their under-ice feeding behaviour and the temporal and spatial overlap between DVM and diel PSD pattern, it is possible that at least from May to July, Antarctic minke whale vocalizations are connected to feeding. This connection could be in a mating context, attracting potential partners to favourable feeding locations (when krill is at the surface, midnight) or simply because feeding on krill at depth during midday limits simultaneous calling.

#### 4.2.4. Leopard seals

Leopard seal vocalizations have been associated with mating: both sexes are known to produce a variety of calls [26]. Owing to their relatively short periods of intense vocal activity, leopard seal vocalizations are a minor source of ambient sound compared with the baleen whale contributions [26]. Along the Weddell Sea coast, leopard seal vocal activity has been observed between December and February and partly followed a diel pattern [26]. This agrees with the timing of the leopard seal contribution recorded at 66° S and 69° S (December to mid-January), although we found no persistent diel pattern in leopard seal PSD.

## 5. Conclusion

**Figure 11.**
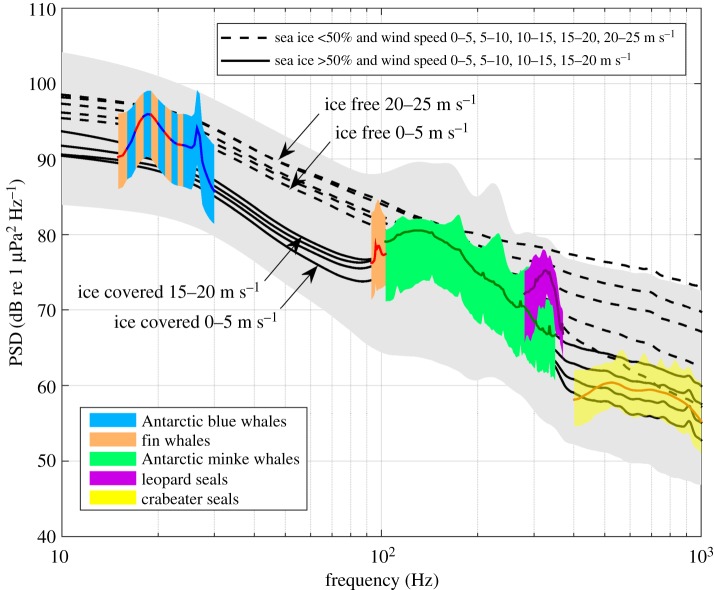
Typical features of the recorded ambient sound at 66° S. Grey area shows range of recorded spectra from 1st till 99th percentile, black lines show the average spectra at different wind speed and sea-ice conditions (averaged within 50 km radius), coloured spectra show averaged marine mammal contribution peaks and the coloured areas the respective 10th and 90th percentile peaks. The blue-red striped area indicates the frequency band between 15 and 25 Hz where the fin and Antarctic blue whale contribution overlap. Spectra have been filtered with running mean window between 5 and 50 Hz. PSD based on factory calibration.

In contrast with the high levels of anthropogenic sound present in the Arctic [9], the Southern Ocean acoustic environment remains largely free of anthropogenic sound and can serve as reference for future ambient sound studies. We recorded substantial natural variability of ambient sound in the Southern Ocean. Our observations show that wind stress, sea ice and marine mammals are the major contributors of ambient sound between 10 and 1000 Hz in the offshore areas of the Southern Ocean. Sea-surface-generated sound dominates the ambient sound spectrum, except for the frequency bands containing MMCs. Figure 11 displays typical ambient sound spectra with respect to the sound sources. The black lines represent averaged spectra during different wind speed and sea-ice conditions. Our results confirm that sea ice reduces ambient sound levels by attenuating surface and acoustic waves, decouples the ambient sound from local wind speed and increases the dominance of distant sources. Southern Ocean ambient sound is strongly connected to the annual cycle: sea-surface-generated sound decreased with growing sea-ice concentration, thickness and extent, and marine mammal vocal activity followed annually reoccurring patterns.

The temporal and spatial variability of the MMCs contains information about behavioural and distribution patterns. Recording MMCs with a higher spatial resolution and combining these measurements with statistical and acoustic propagation models can render it possible to estimate the spatial distribution of vocalizing animals, which will improve our understanding of their behaviour, migration and habitat use.

## Supplementary Material

We added a Pdf document with the supplementary Figures and captions

## Data accessibility

The ambient sound dataset and analysis code are available through the Dryad Digital Repository (http://dx.doi.org/10.5061/dryad.83s93) [65], ERA-interim data through the ECMWF website (http://www.ecmwf.int/), sea-ice concentration data through the University of Bremen (http://www.iup.uni-bremen.de/seaice/amsr/) and sea-ice draught data through the Pangea repository (https://doi.pangaea.de/10.1594/PANGAEA.821257).
