# Supplementary material for: The influence of sea ice, wind speed and marine mammals on Southern Ocean ambient sound
Source: R Soc Open Sci. 2017 Jan 11;4(1):160370. doi: 10.1098/rsos.160370 (PMC5319310; doi:10.1098/rsos.160370)
Supplement: We added a Pdf document with the supplementary Figures and captions [file rsos160370supp1.pdf]

Supplementary Figures related to the paper: The influence of sea ice, wind speed and marine mammals on Southern Ocean ambient sound

Sebastian Menze, Daniel P. Zitterbart, Ilse van Opzeeland and Olaf Boebel

Corresponding author:
Sebastian Menze, Institute of Marine Research, Bergen, Norway (sebastian.menze@imr.no, orcid.org/0000-0002-2680-9794)

Supplementary Figure 1: Schematic representation of the mooring setup used to deploy Aural 66°S	Page 2
Supplementary Figure 2: Schematic representation of the mooring setup used to deploy Aural 69°S	Page 3
Supplementary Figure 3: Comparison of interpolated and measured PSD	Page 4
Supplementary Figure 4: Daily normalized Antarctic minke whale PSD at 66°S over time	Page 5
Supplementary Figure 5: Correlation between long-term spectrograms and sea ice concentration using different averaging radii	Page 5
Supplementary Figure 6: Comparison of long-term spectrogram and average wind speed	Page 6
Supplementary Figure 7: Sound speed and temperature profiles	Page 7

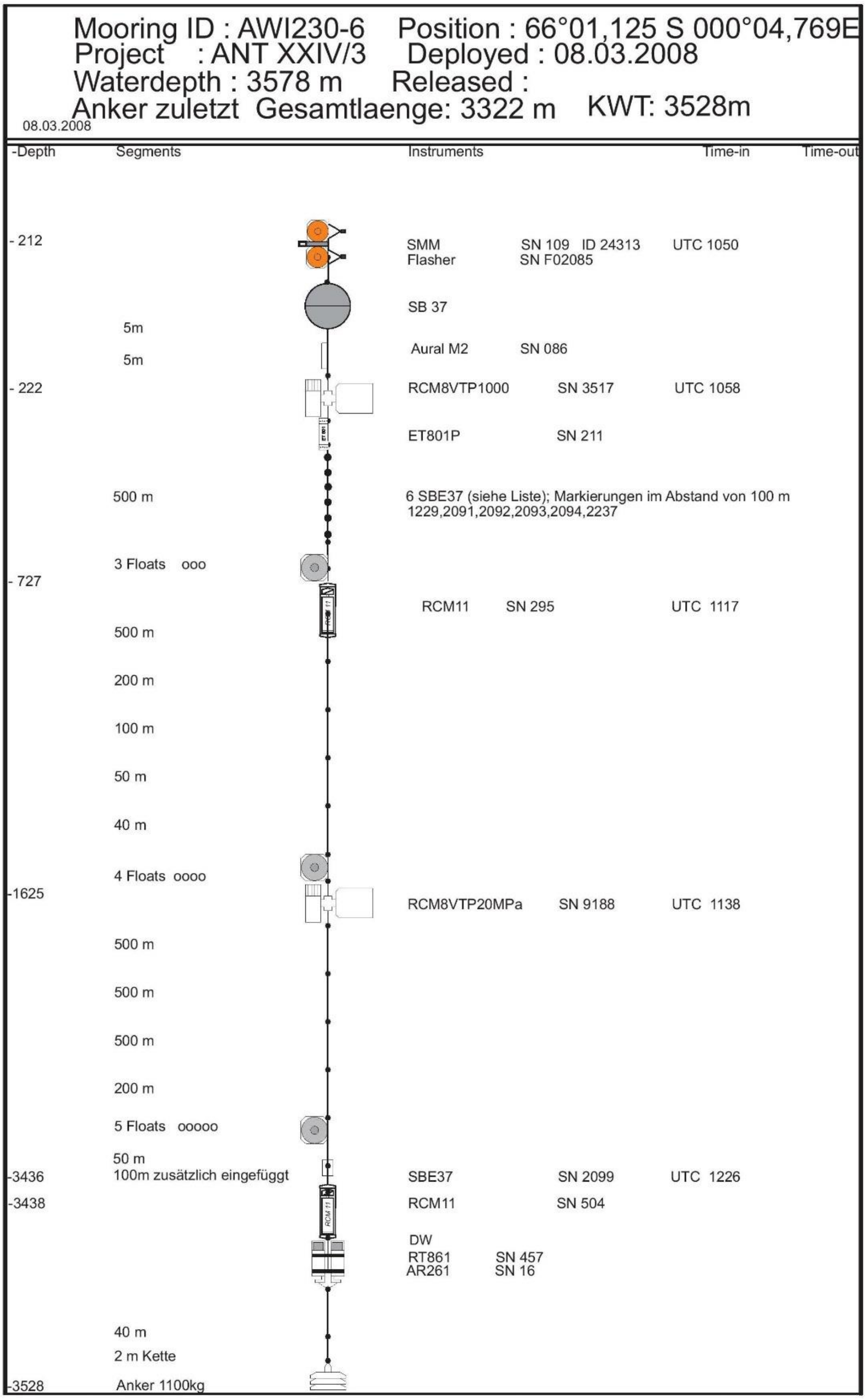

Supplementary Figure 1: Schematic representation of the mooring setup used to deploy Aural 66°S

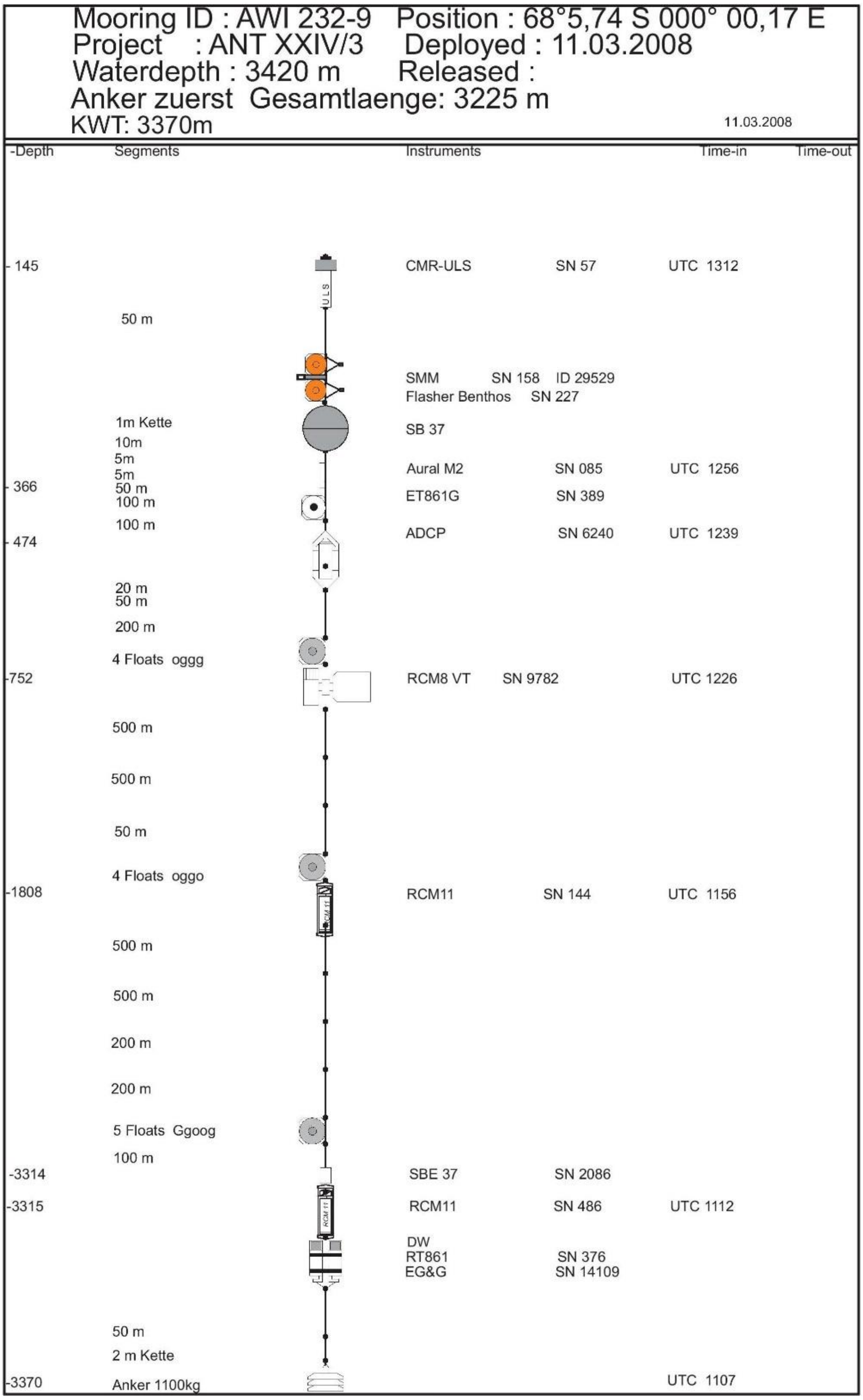

Supplementary Figure 2: Schematic representation of the mooring setup used to deploy Aural 69°S

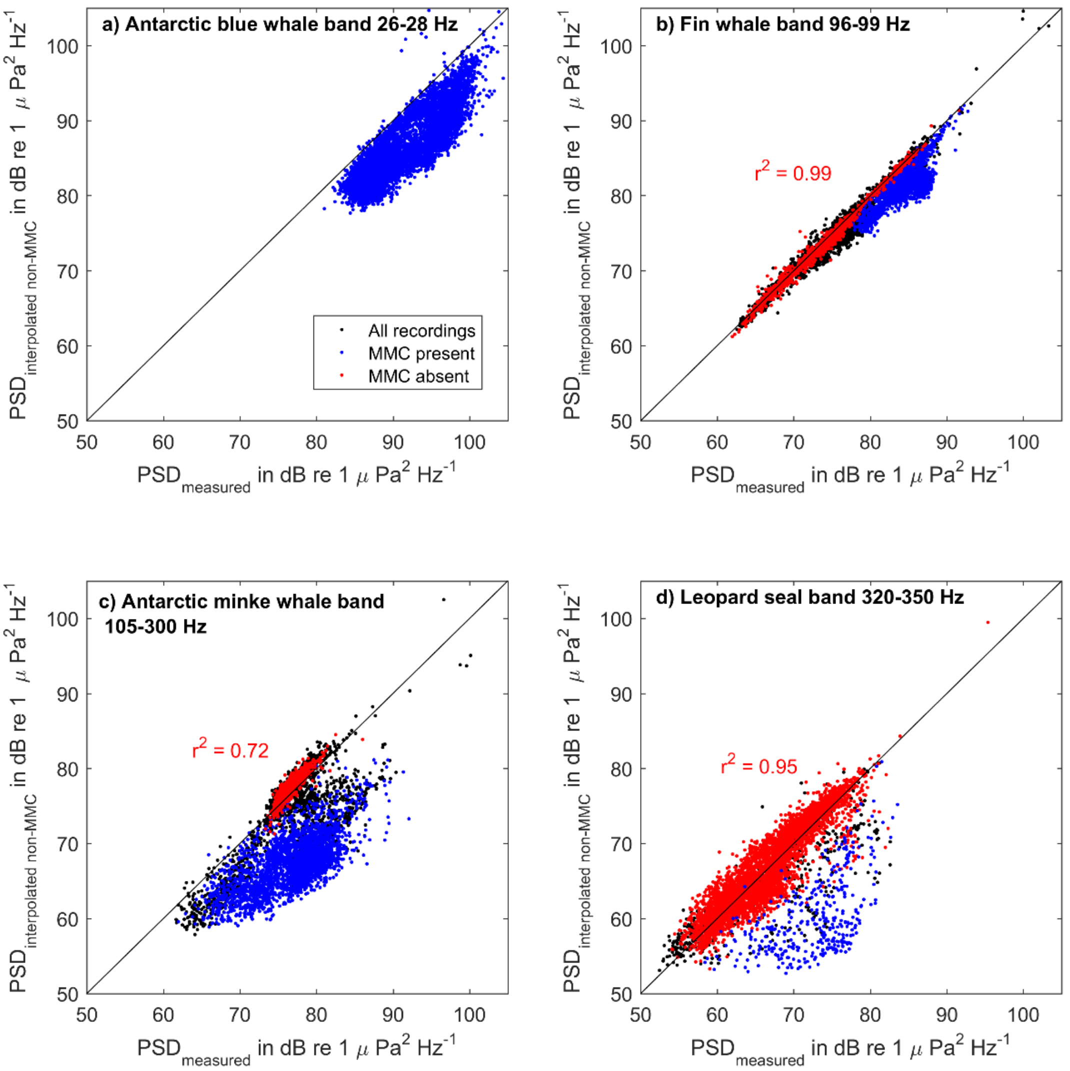

Supplementary Figure 3: Comparison of interpolated and measured PSD at 66°S in the marine mammal contribution frequency bands of a) Antarctic blue whales, b) Fin whales, c) Antarctic minke whales and c) Leopard seals. The black line indicates identical measured and interpolated PSD. Each dot represents a 5-min recordings, blue colour marks spectra where the respective MMC is present and red spectra where the respective MMC is absent.

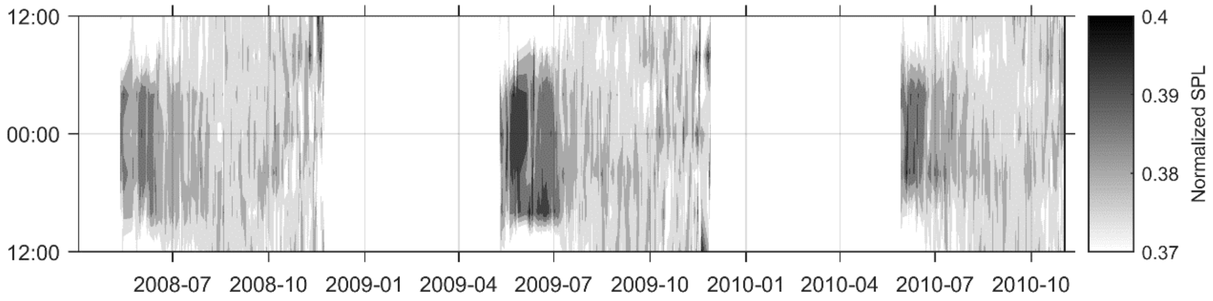

Supplementary Figure 4: Daily normalized Antarctic minke whale PSD at 66°S over time; vertical axis shows time of day (24-h cycle), horizontal axis shows the date and grey scale indicates the daily normalized Antarctic minke whale PSD. From May to July each year the black areas in the middle of the figure indicate a diel pattern, with intense vocal activity at midnight and weak vocal activity during midday. The broad grey areas indicate times where no diel pattern is present in the Antarctic minke whale PSD time series

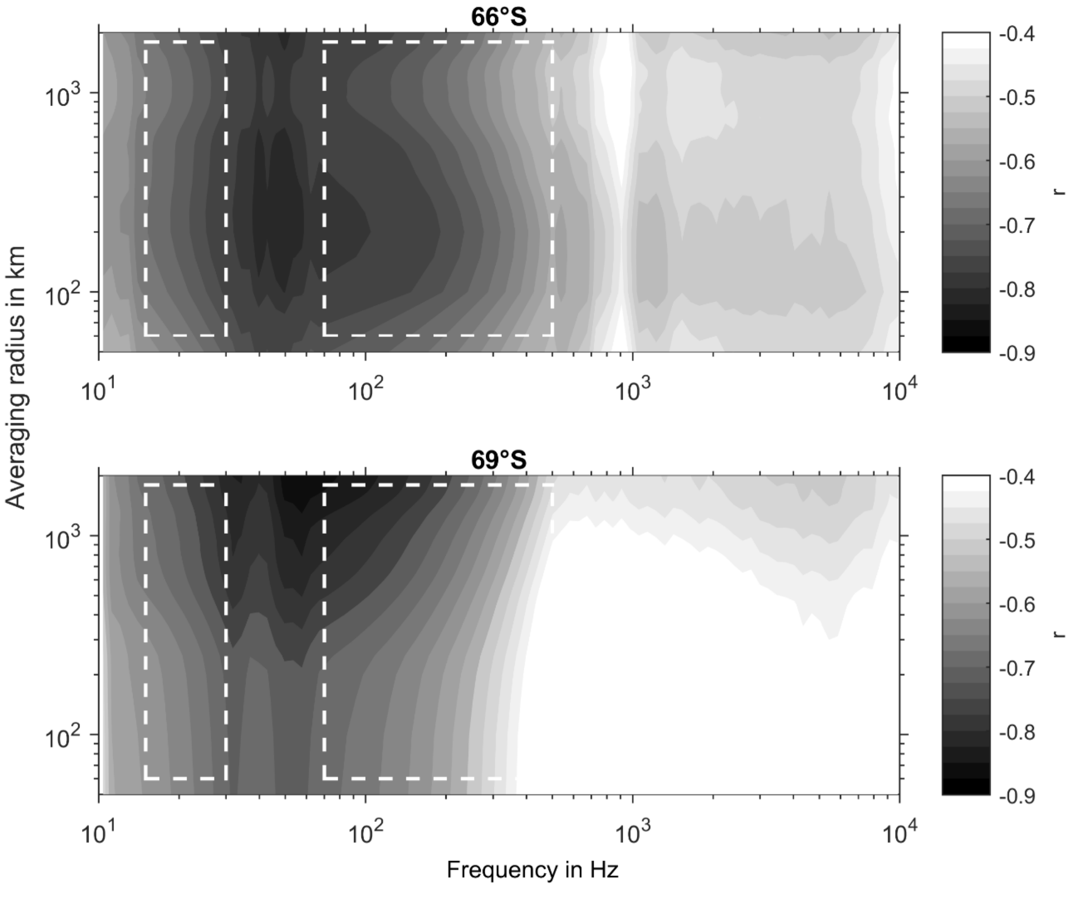

Supplementary Figure 5: Correlation between long-term spectrograms and sea ice concentration using different averaging radii around recorder location from February to July. Horizontal axis shows frequency, vertical axis the averaging radius and grayscale the correlation coefficient. Areas in white dashed box are interpolated correlation coefficients due to interference with marine mammal contributions, upper panel for Aural 66°S, lower for Aural 69°S

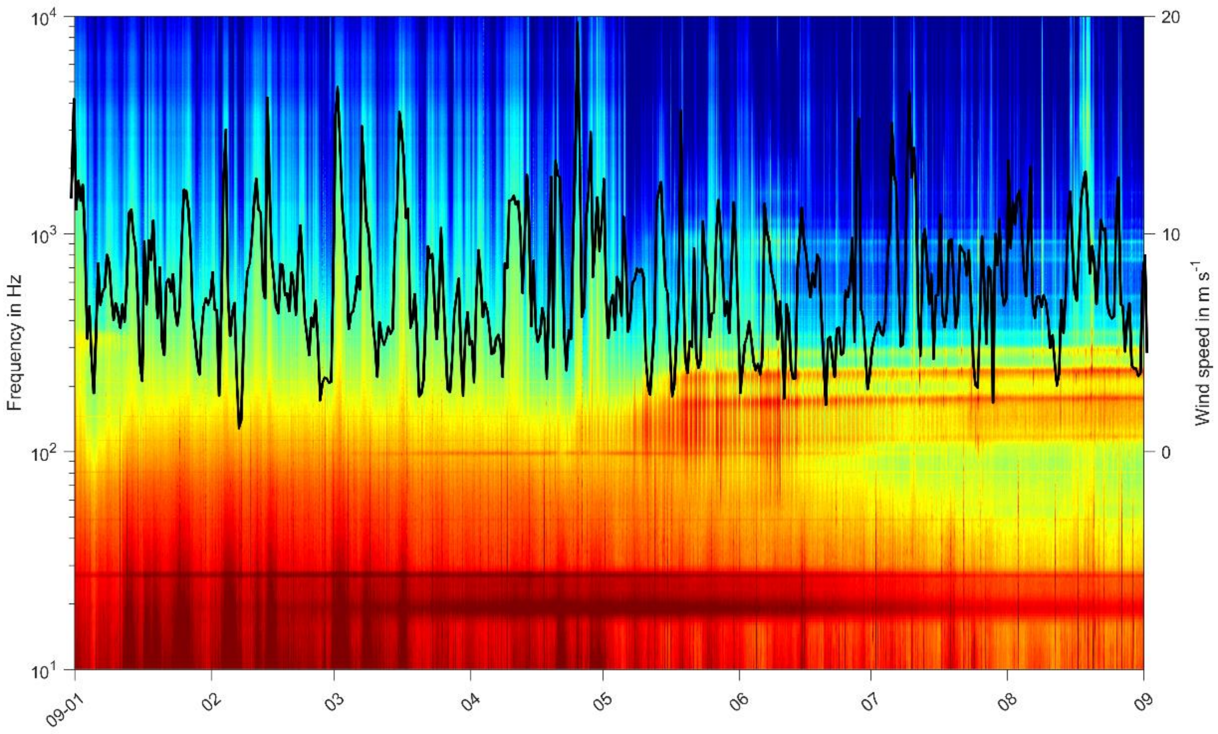

Supplementary Figure 6: Comparison of long-term spectrogram and average wind speed within a 200 km radius (Black line) at 66°S, Colour axis is the same as for Figure 4, ranging from 45 – 100 dB re 1 μPa^2 Hz^{-1}

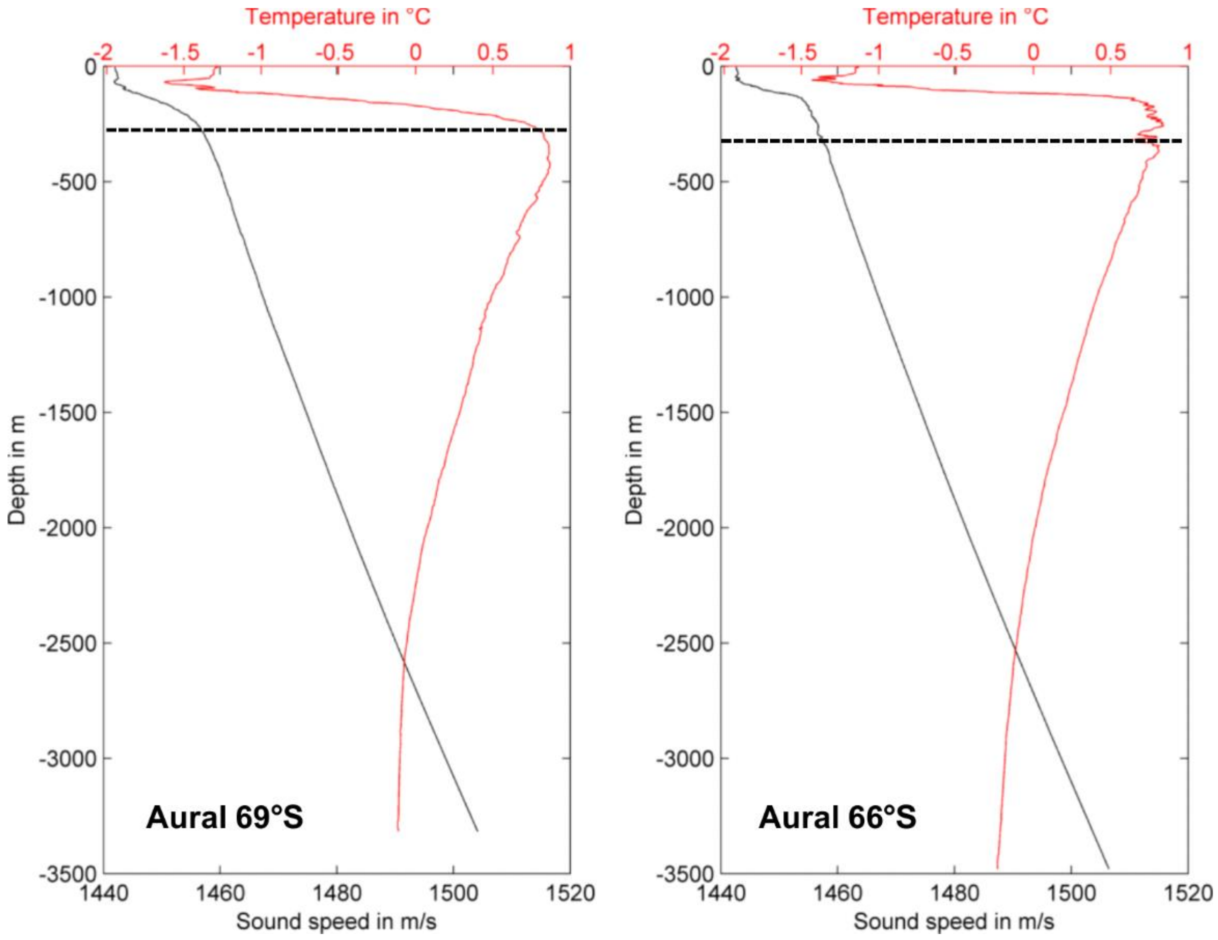

Supplementary Figure 7: Sound speed and temperature profiles measured at the mooring locations during deployment with RV Polarstern, using an SBE911plus CTD system. Dashed line indicates the depth of each recorder.
